# Analysis of tumour ecological balance reveals resource-dependent adaptive strategies of ovarian cancer

**DOI:** 10.1016/j.ebiom.2019.10.001

**Published:** 2019-10-21

**Authors:** Sidra Nawaz, Nicholas A. Trahearn, Andreas Heindl, Susana Banerjee, Carlo C. Maley, Andrea Sottoriva, Yinyin Yuan

**Affiliations:** a Centre for Evolution and Cancer, Institute of Cancer Research, London, UK; b Division of Molecular Pathology, Institute of Cancer Research, London, UK; c Gynaecology Unit, Royal Marsden Hospital, London, UK; d Biodesign Center for Personalized Diagnostics, Arizona State University, Tempe, AZ, USA

**Keywords:** Tumour ecology, Tumour spatial heterogeneity, Cancer evolution, Ovarian cancer, Histological image analysis

## Abstract

**Background:**

Despite treatment advances, there remains a significant risk of recurrence in ovarian cancer, at which stage it is usually incurable. Consequently, there is a clear need for improved patient stratification. However, at present clinical prognosticators remain largely unchanged due to the lack of reproducible methods to identify high-risk patients.

**Methods:**

In high-grade serous ovarian cancer patients with advanced disease, we spatially define a tumour ecological balance of stromal resource and immune hazard using high-throughput image and spatial analysis of routine histology slides. On this basis an EcoScore is developed to classify tumours by a shift in this balance towards cancer-favouring or inhibiting conditions.

**Findings:**

The EcoScore provides prognostic value stronger than, and independent of, known risk factors. Crucially, the clinical relevance of mutational burden and genomic instability differ under different stromal resource conditions, suggesting that the selective advantage of these cancer hallmarks is dependent on the context of stromal spatial structure. Under a high resource condition defined by a high level of geographical intermixing of cancer and stromal cells, selection appears to be driven by point mutations; whereas, in low resource tumours featured with high hypoxia and low cancer-immune co-localization, selection is fuelled by aneuploidy.

**Interpretation:**

Our study offers empirical evidence that cancer fitness depends on tumour spatial constraints, and presents a biological basis for developing better assessments of tumour adaptive strategies in overcoming ecological constraints including immune surveillance and hypoxia.

## Research in context

### Evidence before this study

In high-grade serous ovarian carcinoma there remains a clear need for improved patient stratification, particularly to allow for the identification of patients with higher risk of recurrence. The combination of two measures: genetic diversity of neoplastic cells and that of their surrounding microenvironment, has previously been shown as a promising direction for the further of novel biomarkers. Despite overwhelming evidence on the importance of cancer mutational burden and aneuploidy in eliciting immunoreactivity, other factors shaping this process are elusive.

### Added value of this study

In this study, we demonstrated that the spatial architecture of tumour stroma is a key determinant in successful adaptation of cancer to overcome microenvironmental constraints including immune surveillance and hypoxia, thereby providing new explanations for the lack of specificity in current treatments of ovarian cancer and offering new therapeutic strategies.

### Implications of all the available evidence

The interaction between cancer cells and their surrounding healthy tissue is vital for tumour growth and evolution. In particular, the ecological balance in the vicinity of cancer cells provides critical context to the assumption of different adaptive strategies within cancer cell populations. This study has shown that the clinical consequences of cancer hallmarks, mutagenesis and somatic copy number alterations, also differ under specific tissue ecological contexts. Understanding the biological basis of these fundamentally different tumour ecological contexts could allow for the development of novel targeted therapies, by leveraging the ways in which each context alters the evolutionary trajectory of the disease.

## 1 Introduction

Ovarian cancer is diagnosed in more than 225,000 women per year worldwide and remains a significant cause of gynaecological cancer mortality. High-grade serous ovarian carcinoma (HGSOC) is the most commonly occurring histologic subtype of epithelial ovarian cancer. The majority of women continue to present at advanced stages and the overall 5-year survival rate remains around 40%. The current standard of care for newly diagnosed ovarian cancer is a combination of optimal cytoreductive surgery and platinum-based chemotherapy. The recognition of the importance of homologous recombination deficiency and *BRCA* status in HGSOC is changing clinical practice with the use of PARP inhibitors [1,2]. Despite treatment advances, there remains a significant risk of recurrence at which stage ovarian cancer is usually incurable [3]. The need for improved patient stratification has fuelled the search for new prognostic markers and further subtype identification with the aid of novel molecular signatures [4], [5], [6]. For example, among the four transcriptional subtypes of HGSOC, the mesenchymal subtype with a high amount of infiltrating stromal components such as myofibroblasts and microvascular pericytes was associated with particularly poor survival, and an immunoreactive subtype with favourable outcome [4], [5], [6]. However, clinical prognosticators remain largely unchanged due to the lack of reproducible methods to identify high-risk patients, in particular those who face rapid disease progression despite optimal debulking surgery [7,8].

A promising route for the development of further novel HGSOC biomarkers is to exploit the interplay between cancer cells and their microenvironment, the significance of which in influencing the progression of HGSOC and other cancers has been highlighted extensively [9], [10], [11], [12], [13]. However, microenvironmental influences on neoplastic cells depend on their spatial relationships and specific interactions with multiple types of cells. Therefore, a methodological assessment of this multi-way interplay to gain a system-level, spatially-defined knowledge of the tumour microenvironment, may elucidate the selective advance of genetically heterogeneous cancer cells.

Because the ecology of an organism or cell can be broken down, in the most general terms, into resources and hazards, our recent consensus statements recommended the development of an ecological index that is a composite of both the resources and hazards for neoplastic cells in a tumour [14]. As the first demonstration of such eco-index, this study brings together the concept of ecological habitats defined by a resource-hazard balance in the HGSOC tumour microenvironment to interpret genetic heterogeneity in cancer cells *in situ*. The tumour microenvironment is analogous to the habitat of an organism [15], [16], [17]. Just as an organism must interact with its habitat and other organisms by utilizing resources and evading predators, cancer viability and likelihood of progression are modulated by similar selective pressures in its microenvironment [15]. The theoretical basis for the importance of the microenvironment lies, therefore, in its determination of the resource (R) uptake and predatory hazards (H) for the neoplastic cells [18]. In HGSOC, endothelial cells and fibroblasts in the tumour stroma may support tumour growth by providing growth factors for cancer cells [19], stimulating angiogenesis [20] in order for them to obtain more nutrients, and increasing their invasive potential [21]. On the other hand, high densities of both B cells and the CD8^+^ T-cell infiltrate have been associated with a better prognosis in HGSOC [22,23].

Using automated histology analysis, we recently showed that a high abundance of stromal cells that include fibroblasts and endothelial cells was correlated with poor overall survival, while lymphocytic infiltrate was correlated with favourable prognosis in ovarian cancer [24], consistent with a number of studies [4], [5], [6]. Based on these observations, we propose that stromal cells are associated with the provision of ‘resources’ for cancer while lymphocytic infiltrate is largely a ‘predatory hazard’ in HGSOC. In this study, we: i) define microenvironmental habitats in the spatial surroundings of neoplastic cells, ii) test tumour ecology as a new type of quantitative biomarker for HGSOC and its clinical value independent of known clinical variables in two independent patient cohorts, and iii) present evidence that cancer evolutionary strategy is dependent on the ecological context.

## 2 Materials and methods

### 2.1 Sample set

Samples of treatment-naïve HGSOC tumours from two independent, previously described studies were included as the discovery (*N* = 505, image and genomic data) [6] and validation (*N* = 77, image data only) [24] cohorts. Five-year overall survival rates were available for all patients in both cohorts (Table S1). Patient consent and ethical approval were obtained by the institutional review board of Sun Yat-Sen University Cancer Center and relevant institutional review boards participated in the TCGA study. Patient selection criteria included Federation of Gynecology and Obstetrics (FIGO) stage III and IV disease, a lower bound of 0.3 mm^2^ for the sample area consisting of tumour, availability of H&E-stained histological images of surgically resected tumours and exclusion of TCGA samples recently re-classified as non-HGSOC [25]. In the discovery cohort (*N* *=* 505, 967 frozen sections, 469 patients with two sections each and 36 patients with one section each), available clinical risk factors included debulking status (defined as ‘optimal’ if no residual disease larger than 1 cm is present after surgical cytoreduction and ‘sub-optimal’ otherwise) (*N* = 459); disease stage; patient age; radiological response to primary treatment (RECIST 1.1 criteria) (*N* = 360), and *BRCA1/2* mutation (*N* = 282), taken as a germline or somatic mutation in either *BRCA1* or *BRCA2*. Additionally in the discovery cohort, mutational burden scores were available for *N* = 287 patients; arm/chromosomal SCNA, defined as the rate of whole arm or chromosome amplification/deletion in a sample, for *N* = 481 patients, directly downloaded from [34]; number of telomeric allelic imbalances (NtAI), defined as the number of sub-telomeric regions with allelic imbalance that start beyond the centromere and extend to the telomere, for *N* = 456 patients; large-scale transitions (LST), defined as the number of chromosomal breaks between adjacent regions of at least 10Mb for *N* = 456 patients; loss of heterozygosity (HRD-LOH), defined as the number of regions with LOH which are larger than 15Mb but shorter than the whole chromosome, for *N* = 456 patients; and mRNA expression profiles for *N* = 454 patients. Available clinical data for all patients in the validation cohort (*N* *=* 77, one FFPE section per patient) included clinical factors such as tumour debulking status, disease stage, and serum CA125 level and patient age at diagnosis. Additionally for four tumours in the validation cohort, anti-VEGF antibody-stained serial sections were also available. All patients received platinum-based chemotherapy, and 94% in the discovery cohort and 88% in the validation cohort also received a taxane.

### 2.2 H&E image analysis

Whole-tumour section images were processed using a previously published image analysis pipeline [24] to detect individual cancer, lymphocyte and stromal cell nuclei. Additional extension to this pipeline including staining normalization [26] was used for accommodating the staining variability presented in TCGA images. Cell classification was performed using a support vector machine with 97 morphological and textural features derived from haematoxylin positive nuclei. Validation of the automated image analysis was based on experiments involving five orthogonal data types [27]. First, the balanced accuracy as an average of sensitivity and specificity of our classifier was evaluated and found to be 80.6% for stromal cells, 85⋅0% cancer cells and 82⋅6% lymphocytes (based on a set of *N* *=* 894 hand-annotated single cell nuclei). Second, we found that automated cell scoring using our pipeline was highly correlated with independent scoring provided by TCGA pathologists (Fig. S1). Third, using gene expression data and enrichment analysis [28], [29], [30], we identified significant associations between cell abundance and relevant functional pathways and biological processes including cell cycle and checkpoints for cancer cells, chemokine and leukocyte transendothelial migration for lymphocytes, and matrisome and collagen formation for stromal cells, supporting the biological relevance of automated image analysis results. Fourth, tumour purity measures using the gene expression-based method ESTIMATE [31] and copy number-based method ABSOLUTE [32] correlated with tumour cellularity, defined as the cancer cell abundance estimated from the image analysis, (ESTIMATE *r* = 0⋅44, *P* < 0⋅001; ABSOLUTE, *r* = 0⋅43, *P* < 0⋅001). Finally, immunohistochemistry sections of cancer, lymphocyte and stromal markers (CK7, CD3, and SMA) showed good concordance with H&E image analysis results (Supplementary Methods).

### 2.3 Local ecological habitat classification and robustness

Following image analysis to obtain cell location data, a grid of squares of width 100 μm (20 pixels with a resolution of 5 μm/pixel) was applied to each image. Squares were classified into different ecological habitats based on the abundance of lymphocytes and stromal cells relative to the number of cancer cells within the square (Fig. S2, Materials and Methods). For example, a square with a high abundance of both stromal cells and lymphocytes was deemed a ‘high resource-high hazard’ habitat. The threshold for defining high and low abundance of resource/stromal cells for each square *i* was defined as follows:

(1) $$th_{str}^{i}=\;k_{str}\times \;n_{c}^{i}$$

where $th_{str}^{i}$ is the stromal cell threshold for square *i*, $n_{c}^{i}$ is the number of cancer cells in square *i* and *k_str_* is a constant. Similarly, the threshold $th_{lym}^{i}$ for hazard/lymphocytic abundance for each square *i* was determined by:

(2) $$th_{lym}^{i}=\;k_{lym}\times \;n_{c}^{i}$$

where *k_lym_* is also a constant. The two constants *k_str_* and *k_lym_* were chosen following random sampling of 100 squares from 100 randomly selected tumours in the discovery cohort. From an ecological perspective, the number of individuals in a trophic level generally decreases with trophic level. Applying this concept to lymphocytes as predators and cancer cells as prey, it follows that there would typically be fewer lymphocytes, even within a high hazard habitat, than the number of stromal cells in a high resource habitat. Consequently, the value selected for *k_lym_* should be higher than that of *k_lym_*. In this work, a *k_lym_* of 0⋅2 was chosen, which appeared to split the habitats into two approximately equal groups (Fig. S2A).For stromal resource, a *k_str_* of 0⋅5 was selected. It was observed that this threshold appeared to split the sample set such that approximately 25% of the habitats were defined as high resource (Fig. S2B). Since some noise can be expected in the data due simply to the presence of stromal cells along the tumour margin as part of the body's natural response to a tumour, and thus not a manifestation of cancer-stromal crosstalk that benefits cancer cells, a high *k_str_* ensured this was minimized while still returning two sizeable groups of habitats defined by relative stromal abundance. For the 469 patients in the discovery cohort for whom two histological sections per tumour were available, the total number of each habitat type as a weighted average from the two sections was used.

### 2.4 Spatial stability and scale tolerance of local EcoScore

To test the spatial stability of EcoScore, we first introduced incremental grid shifts, computed EcoScore and compared to the original data, since slight shifts in the grid placement may result in changes to the EcoScore value. We implemented shifts of 5, 10 and 15 pixels (25 μm, 50 μm and 75 μm respectively) in both *x* and *y* in the original grid of squares of side length 20 pixels (100 μm), for all samples in the validation cohort. In each case, the 40th percentile was used to dichotomize continuous EcoScore values into high and low, as in the original data. None of the 77 patients changed EcoScore groups following any of the grid shifts. The mean EcoScore was also computed after every grid shift, and in all cases the change was less than 2% of the original value.

Next, we tested the spatial scale tolerance of local EcoScore by varying the size of grid squares. We tested squares of side lengths 10, 40, 60 and 100 pixels (50 μm, 200 μm, 300 μm and 500 μm respectively), and compared patient stratification at 40% to the original data derived using 20 pixel (100 μm) squares. For 40–60 pixels, only 3 patients changed groups, but this increased to 6 patients at 10 pixels and 100 pixels, thus making the 20–60 pixel range the most stable for evaluating EcoScore. To maximize the spatial resolution of our method, we opted for the smallest square size in this range, 20 pixels. Further details on robustness testing are given in the Supplementary Sweave.

### 2.5 Global ecological balance

To explore wide-ranging ecological impacts from resources and hazards for cancer in the tumour microenvironment, we computed a global EcoScore for every sample as follows. First, hotspot analysis [33] was applied to each whole-tumour section to identify statistically significant spatial clustering in lymphocytes and stromal cells, as described in Supplementary Methods. Next, for each habitat in the tumour, distances to the nearest lymphocyte and stromal cell hotspot were computed. The global EcoScore was then defined as:

(3) $$\text{Global}\;\text{EcoScore}=\frac{\sum_{i}^{n}d_{i}^{lym}}{\sum_{i}^{n}d_{i}^{str}},$$

where *n* is the total number of habitats in the tumour section, and $d_{i}^{lym}$ and $d_{i}^{str}$ represent the nearest distance to a lymphocyte and stromal cell hotspot respectively for habitat *i.* It is effectively a ratio of the mean distance to a lymphocyte and stromal cell hotspot for a habitat in the sample, and higher values indicate greater relative proximity to resource-rich regions.

### 2.6 Survival analysis

To test the association of the local and global EcoScore with five-year overall survival, we constructed univariate and multivariate Cox proportional hazards models for the discovery and validation cohorts. Every 5th percentile in the range 15–85% of the EcoScore was evaluated as a dichotomizing threshold in a univariate Cox model in the discovery cohort. Patients were split into two groups based on this threshold and their survival rates compared. A log rank *P* value of less than 0⋅003 (α = 0⋅05 corrected for multiple testing using the conservative Bonferroni method) was taken as a significant result. Where multiple thresholds with a corrected p value less than 0⋅003 were found, the threshold with the lowest *P* value was selected. Significant ecological factors were assessed in the validation cohort by dichotomization at the same percentile.

### 2.7 Other statistical methods

The two-sided Jonckheere trend test was used to determine association between a continuous variable and an ordered categorical variable with more than two categories, whereas for a variable with two categories, ordered or otherwise, a two-sided Wilcoxon rank sum test was used. The Spearman rank correlation test was used to test for association between two continuous variables.

## 3 Results

### 3.1 Automated histology image analysis enables spatial mapping of ecological habitats for HGSOC tumours

**Fig. 1:**
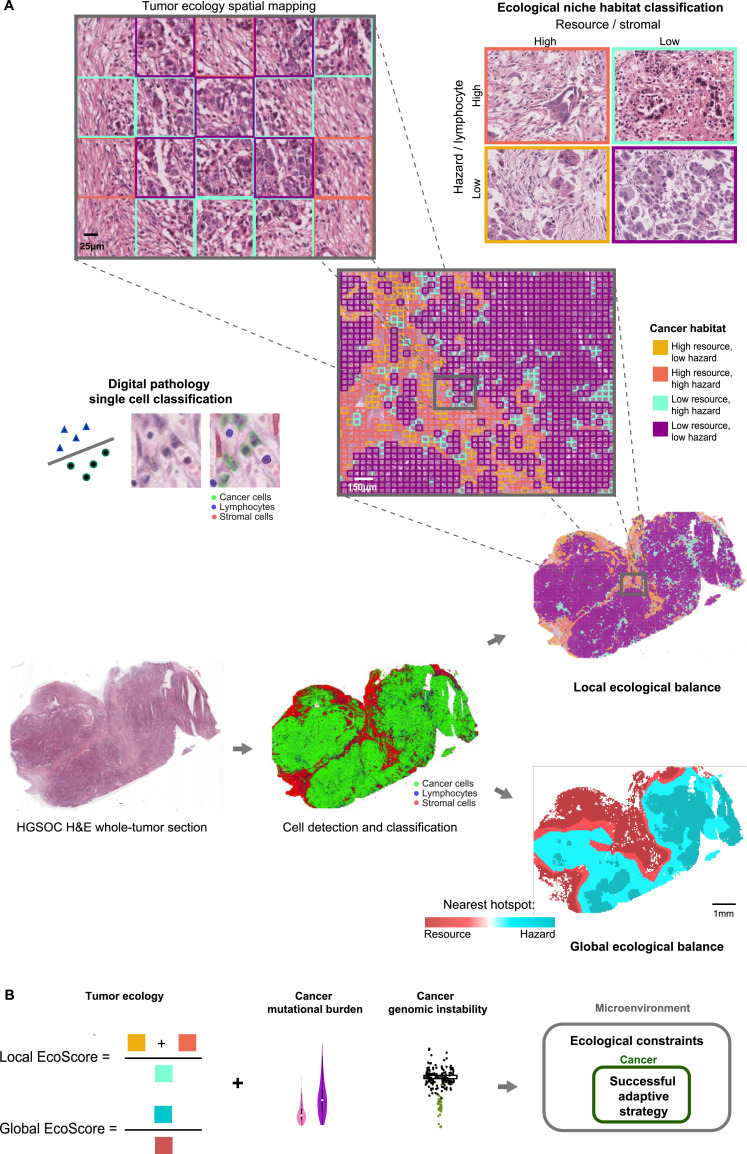
An ecological resource-hazard characterization of high-grade serous ovarian carcinoma (HGSOC). (**A**) Our H&E image analysis pipeline, based on 104 quantitative measures of cell nucleus texture and morphology to classify nuclei into cancer, lymphocyte and stromal cell nuclei and provides their spatial coordinates, is used to process an H&E-stained ovary tumour sample (left) and map the distribution of the three cell types (middle). The sample is then divided into small regions using a grid of square size 100 μm. The abundance of lymphocytes and stromal cells is evaluated in each square that contains at least one cancer cell to determine its local ecological habitat type. Spatial distributions of the four habitats in this tumour are shown in a heatmap (right, top) with colours corresponding to the example habitat images above. Also shown is the grid-based habitat classification in one region of the tumour sample. In addition, hotspot analysis is performed to detect spatial clustering in lymphocytes and stromal cells in each sample. The distances to the nearest lymphocyte and stromal hotspots are evaluated for every habitat type and indicated in the heatmap (right, bottom). This is used to determine the global ecological balance in a tumour Based on previous findings, we propose that stromal cells act as ‘resources’ for cancer while lymphocytes present a predatory ‘hazard’. (**B**) The local EcoScore is computed as the balance between tumour-promoting and tumour-inhibiting habitats, and the global EcoScore is the ratio of the mean distances to the nearest lymphocyte and stromal cell hotspots for a habitat in the sample. Together with genomic tumour analyses, we present an integrated characterization of HGSOC.

**Fig. 2:**
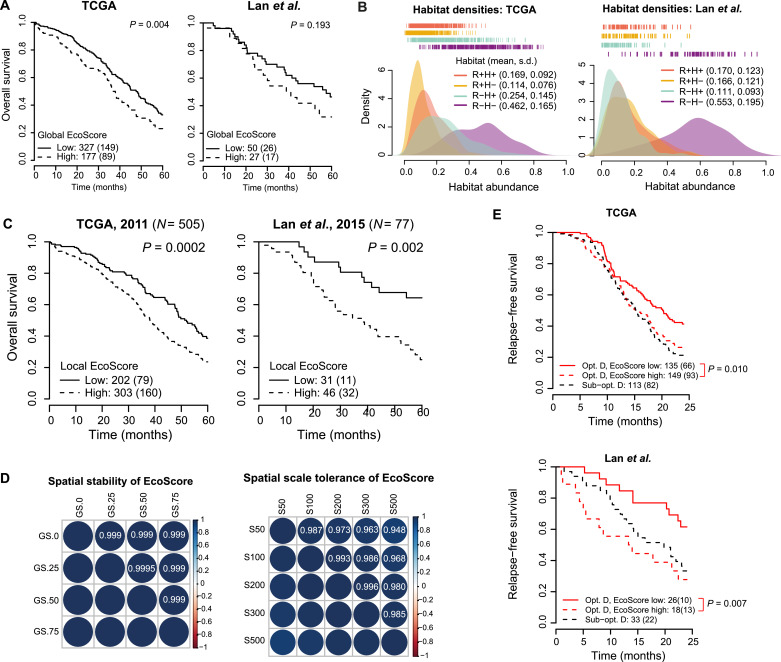
Assessing the prognostic value and stability of EcoScore. (**A**) Kaplan-Meier plots display the prognostic value of global EcoScore, defined by the mean ratio of distance to the nearest lymphocyte and stromal cell hotspots for a cancer habitat, for 5-year overall survival in two independent cohorts of HGSOC patients. Numbers outside parentheses indicate group size and numbers inside parentheses indicate deaths. (**B**) Kernel density plots show the distribution of abundances of the four ecological habitats in the TCGA (left) and Lan et al. (right) cohorts. Mean and standard deviation (s.d.) of each distribution are given in brackets in the legends. In both cohorts, the peak of the R − H− distribution is shifted to the right of the others and the s.d. of this distribution is highest. (**C**) Kaplan-Meier plots display the prognostic value of local EcoScore, defined by the balance of tumour-promoting habitats against tumour-inhibiting habitats, for 5-year overall survival in two independent cohorts of HGSOC patients. Numbers outside parentheses indicate group size and numbers inside parentheses indicate deaths. (**D**) Top: Heatmap illustrating the correlations between local EcoScore computed for each sample in the validation cohort following grid shifts of 0 (original), 25, 50, and 75 μm. Each square in the grid is 100 μm. Bottom: Heatmap illustrating the correlations between EcoScore computed for each sample in the validation cohort using grid square sizes of 50, 100 (original), 200, 300, and 500 μm. The resulting values of EcoScore are highly correlated. (**E**) Kaplan-Meier curves display stratification for overall survival using local EcoScore of patients who had optimal debulking surgery (Opt. debulking, red curves) in the discovery and validation cohorts. Patients who had optimal debulking surgery and a high tumour EcoScore had a statistically similar overall survival probability compared to patients who had sub-optimal debulking (Sub-opt. debulking, black dashed curves).

A total of 1051 haematoxylin and eosin (H&E)-stained, whole-tumour section images from 582 HGSOC patients from two independent cohorts were processed with our computational pipeline for automated detection of cancer, lymphocyte and stromal cell nuclei (Materials and Methods). In both cohorts we observed an overall pro-tumour effect of stromal cells and an anti-tumour effect of lymphocytes (Fig. S3, Table 1, Materials and Methods). Therefore, stromal cells could be responsible for the provision of ‘resources’ for cancer while lymphocytic infiltrate may largely be a ‘predatory hazard’ in HGSOC. Based on this assumption, tumour regions containing cancer cells were classified into one of four ecological habitat types: R + H+ (high resource-high hazard), R + H- (high resource-low hazard), R − H− and R − H+ (Fig. 1). The four habitats were defined by the abundance, relative to and in the vicinity of cancer cells, of stromal cells as resource and lymphocytes as predatory hazard for cancer (Materials and Methods, Fig. 2A). Further, we identified statistically significant hotspots of resources and hazards in each sample (Materials and Methods), and computed the shortest distance to each of these for every habitat in the sample.

**Table 1:** Univariate and multivariate 5-year overall survival analyses results in two independent cohorts of HGSOC patients with FIGO stage III and IV disease. The univariate prognostic values of clinical risk factors and cell abundance measures, where available, in the two cohorts are listed, as are those of our ecological measures found to be prognostic in both the discovery (TCGA, 2011) and validation (Lan et al., 2015) cohorts: abundance of the high resource-low hazard (R + H−) habitat and the EcoScore. The independence of our ecological measures as prognostic markers was evaluated in a multivariate model including risk factors found to be prognostic in the univariate setting in the given cohort. For the discovery cohort, this included age at diagnosis, *BRCA1/2* mutation, response to primary chemotherapy and lymphocytic and stromal cell abundance; for the validation cohort, this included stromal cell abundance and CA125 level at diagnosis. In the validation cohort, CA125 levels were obtained from blood; in the discovery cohort, data for serum CA125 level at diagnosis were unavailable, hence the expression of *MUC16*, the gene encoding CA125, was used. Results for ecological measures found to be prognostic in univariate and multivariate analyses in both cohorts are shaded. HR: hazard ratio; CI: confidence interval; *N*: total number of patients in the cohort included in this study; AU: arbitrary units; p: log rank *P* value (*p*<0⋅003 has been interpreted as a significant result).

		Discovery, TCGA, *N* *=* 505		Validation, Lan et al., *N* *=* 77	
		HR (95% CI)	p	HR (95% CI)	p
**Univariate**	**Local EcoScore**	1⋅65 (1⋅26–2⋅16)	0⋅0002	2⋅91 (1⋅46–5⋅79)	0⋅0015
	**Global EcoScore**	1⋅47 (1⋅13–1⋅92)	0⋅0037	1⋅50 (0⋅81–2⋅77)	0⋅193
	**R** **+** **H+ habitat**	0⋅79 (0⋅54–1⋅09)	0⋅137	1⋅14 (0⋅48–2⋅69)	0⋅773
	**R** **+** **H**− **habitat**	1⋅64 (1⋅26–2⋅13)	0⋅0002	2⋅44 (1⋅33–4⋅50)	0⋅003
	**R** − **H+ habitat**	0⋅58 (0⋅45–0⋅76)	<0⋅0001	0⋅78 (0⋅43–1⋅43)	0⋅424
	**R** − **H**− **habitat**	1⋅42 (1⋅06–1⋅89)	0⋅017	0⋅57 (0⋅31–1⋅07)	0⋅076
	**Stromal cell abundance**	1⋅40 (1⋅05–1⋅85)	0⋅020	2⋅41 (1⋅19–4⋅91)	0⋅012
	**Lymphocytic abundance**	0⋅66 (0⋅51–0⋅85)	0⋅001	0⋅85 (0⋅47–1⋅56)	0⋅606
	**Tumour debulking status**	1⋅19 (0⋅90–1⋅57)	0⋅230	1⋅75 (0⋅96–3⋅18)	0⋅065
	**Age at diagnosis**	1⋅02 (1⋅01–1⋅04)	<0⋅0001	1⋅03 (1⋅00–1⋅07)	0⋅053
	**Disease stage**	1⋅32 (0⋅94–1⋅84)	0⋅108	1⋅26 (0⋅49–3⋅19)	0⋅631
	***BRCA1/2* mutation**	0⋅53 (0⋅35–0⋅82)	0⋅0035	–	–
	**CA125 level at diagnosis/*MUC16* expression (AU)**	1⋅03 (0⋅95–1⋅12)	0⋅461	1⋅00 (1⋅00–1⋅00)	0⋅003
	**Response to primary treatment**				
	Complete response	0⋅27 (0⋅20–0⋅37)	<0⋅0001	–	–
	Partial response	2⋅54 (1⋅75–3⋅68)	<0⋅0001		
	Stable disease	1⋅67 (0⋅93–3⋅02)	0⋅084		
	Progressive disease	4⋅53 (2⋅93–7⋅00)	<0⋅0001		
Multivariate	**Local EcoScore**	2⋅15 (1⋅15–4⋅01)	0⋅016	3⋅22 (1⋅27–8⋅15)	0⋅014
	**R** **+** **H− habitat**	1⋅28 (0⋅78–2⋅11)	0⋅326	1⋅12 (0⋅52–2⋅45)	0⋅770
	**Stromal abundance**	0⋅98 (0⋅55–1⋅75)	0⋅953	1⋅08 (0⋅42–2⋅79)	0⋅871
	**Immune abundance**	0⋅69 (0⋅41–1⋅15)	0⋅151	–	–
	**CA125 level at diagnosis**	–	–	1⋅00 (1⋅00–1⋅00)	0⋅006
	**Age at diagnosis**	1⋅02 (1⋅00–1⋅03)	0⋅117	–	–
	***BRCA1/2* mutation**	0⋅57 (0⋅35–0⋅92)	0⋅023	–	–
	**Response to primary treatment**	0⋅22 (0⋅15–0⋅34)	< 0⋅0001	–	–

### 3.2 The EcoScore is a strong prognostic factor for overall survival

We combined the ecological habitats into a single local ecological score for a tumour, the local ‘EcoScore’, that balances pro-tumour habitats against tumour-inhibiting habitats. We hypothesized that the R +H− habitat presents the most permissive environment for cancer cells and therefore plays a tumour-promoting role, and that the R − H+ habitat will be primarily tumour-inhibiting. The R + H+ habitat will favour cancer cells capable of immune evasion that thereby profit most from the abundant resources. Hence, according to life history theory(18), we proposed this habitat to be favourable for cancer cells. To systematically measure the ecological influences occurring at the tumour-stroma interface, we defined the local EcoScore as follows: $\text{EcoScore}=\;\frac{R+H+\;+\;R+H-}{R-H+}.\;$It should be noted that R − H− habitats, the most abundant habitat in each sample (Fig. 2B), have been excluded from the local EcoScore formula. This is because the majority of the R − H− habitats were found in tumour nests containing very few non-tumour cells (Fig. S4A) and are thus less relevant for cancer-host cell interaction. Furthermore, no clear between the abundance of R − H− habitats and overall survival relationship was found across the two patient cohorts (Fig. S4B).

In addition, to test wide-ranging ecological effects from resources and hazards, we computed a global EcoScore by taking the ratio of the mean shortest distances to lymphocyte and stromal cell hotspots for a habitat in the sample (Materials and Methods). We hypothesized that a higher value, indicating relatively greater proximity to resource-rich regions, would be associated with lower overall survival.

A high local EcoScore was associated with poor overall survival in both the discovery and validation cohorts (Fig. 2C). The effect was independent of known risk factors for HGSOC, including debulking status, patient age, disease stage, *BRCA* mutation, serum CA125 level/gene expression, response to primary treatment, and stromal and lymphocytic abundance (defined as the fraction of all detected cells that are stromal cells or lymphocytes respectively) (Table 1). Moreover, among the microenvironmental measures including cell and habitat abundances, only local EcoScore and R + H− abundance were prognostic in both cohorts following an optimal threshold search in the discovery cohort and correction for multiple testing (Fig. 2C, Table 1). However, local EcoScore, but not R + H− abundance, was prognostic independently of the above risk factors, despite its correlation with lymphocytic and stromal cell abundance (Fig. S5A–B, Table 1). In a multivariate model in the discovery cohort consisting of all available risk factors for HGSOC as well as local EcoScore and cell and habitat abundance measures found to be prognostic in the cohort, the local EcoScore was the only measure to provide independent prognostic information (Fig. 2C). Spatial stability and scale tolerance of local EcoScore were demonstrated through tests over a range of parameters in its spatial configuration (Materials and Methods, Fig. 2D). Comparison with molecular subtypes that have been associated with the tumour microenvironment [5], the mesenchymal molecular subtype was found to be enriched in the high local EcoScore tumours (*P* < 0.0001), whereas, Low local EcoScore tumours had a significantly higher proportion of the immunoreactive subtype (*P* < 0⋅0001, Fig. S6A), consistent with their prognostic implications. When compared with global EcoScore that was also prognostic for poor survival in the discovery cohort (*P* *=* 0.0037, Table 1), local EcoScore had a much higher prognostic power. Furthermore, global EcoScore was not prognostic in the validation cohort (*P* > 0⋅05, Table 1), thus the local EcoScore (henceforth referred to as EcoScore) was selected for further analyses as the stronger indicator of ecological balance in the tumour microenvironment.

To assess further clinical relevance of the EcoScore, we tested its prognostic value in patients who had optimal tumour debulking surgery. In both cohorts, patients who had optimal debulking surgery could be stratified into low and high risk groups for relapse-free survival using the EcoScore, which identified a subgroup with a significantly higher risk of relapse within two years (Fig. 2E). Patients who had optimal debulking surgery but with a high EcoScore had similar relapse-free survival to patients who had sub-optimal debulking surgery (*P* > 0.05). However molecular subtypes did not stratify patients who had optimal debulking surgery in the TCGA cohort (*P* > 0⋅05, Fig. S6B), although the mesenchymal subtype has been previously associated with a lower rate of optimal debulking surgery [5].

### 3.3 Tumour EcoScore and mutational burden co-define a strong prognostic model

**Fig. 3:**
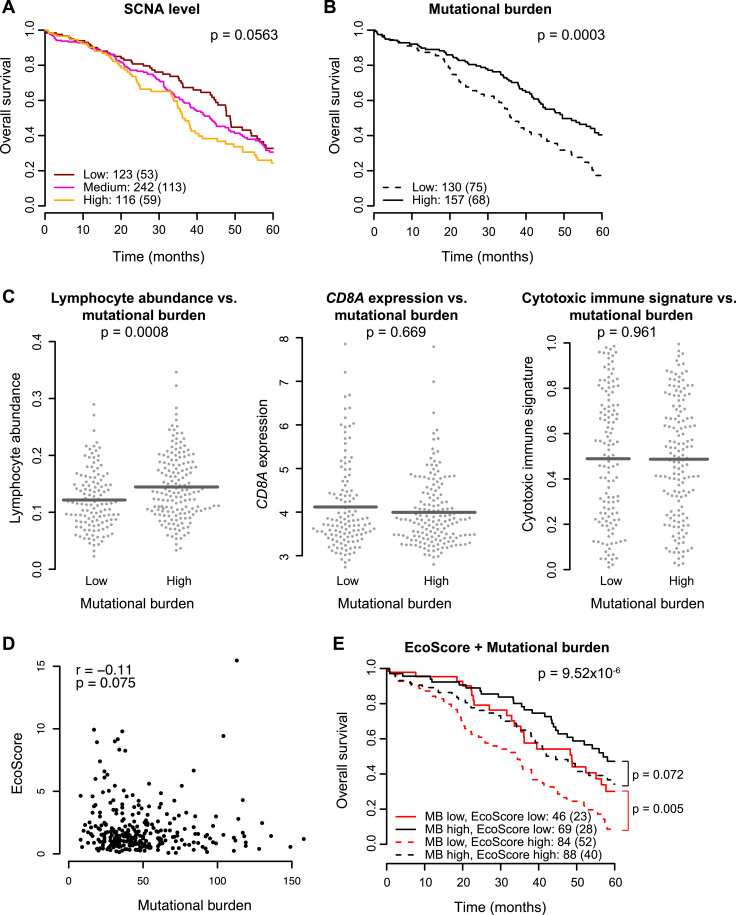
Integrating histology-derived ecological measures with cancer genetics. (**A**) Kaplan-Meier curves show SCNA level, defined by the rate of whole arm or chromosomal amplification/deletion and available for *N* *=* 481 patients in the discovery cohort, is not prognostic. Low, medium and high are defined by the 25th and 75th percentiles used as cut-offs. (**B**) Kaplan-Meier curves show the prognostic value of mutational burden (MB), defined by the total number of somatic, non-synonymous mutations identified in the tumour, available for 287 patients in the discovery cohort. Low and high are defined by the 45th percentile used as the cut-off. Numbers outside parentheses indicate group size and numbers inside parentheses indicate deaths. (**C**) Swarm plots show the distribution of lymphocyte abundance (left), *CD8A* expression (middle) and the cytotoxic immune signature proposed by Davoli et al., 2017 (right), in patients grouped by mutational burden. (**D**) Scatter plot displays the lack of correlation between mutational burden and EcoScore in the discovery cohort. (**E**) A prognostic model combining EcoScore and mutational burden offers substantial improvement in patient stratification compared to these measures used individually. Patients with a low mutational burden and a high EcoScore (dashed red line) faced the poorest survival rate. Numbers outside parentheses indicate group size and numbers inside parentheses indicate deaths.

To identify the most relevant indicators of HGSOC ecology and evolution, we first tested the prognostic value of different genomic measures in the TCGA cohort, including mutational burden, defined as the number of non-synonymous somatic mutations; total arm and whole-chromosome somatic copy number alterations (SCNA); [34] number of telomeric allelic imbalances (NtAI); [35] large-scale transitions (LST) [36], and loss of heterozygosity (HRD-LOH) [37] (Supplementary Methods). Focal somatic copy number alterations [34] was correlated with mutational burden (*r* = 0⋅42, *P* < 0⋅0001), hence it was excluded from this analysis. SCNA, NtAI, LST and HRD-LOH were not found to be prognostic for overall survival (all *P* > 0⋅05, Fig. 3A, Table S2). However, a high mutational burden was associated with better prognosis and high lymphocytic abundance based on H&E image analysis (Fig. 3B-C; TCGA cohort 1: *P* = 0⋅006; TCGA cohort 2: *P* = 0⋅005, Fig. S7A), consistent with the notion of cytotoxic immune response to tumour neoantigen [38], [39], [40]. None of the genomic measures were found to correlate with tumour cellularity, calculated as the proportion of cells within a tumour region that are tumour cells, therefore this association between mutational burden and lymphocytic abundance cannot be explained by abundance of non-tumour cells (all *P* > 0⋅05, Fig. S7B). In comparison, *CD8A* expression and the cytotoxic immune signature [34,41] were not associated with mutational burden (Fig. 3C). EcoScore was not correlated with mutational burden (*P* > 0⋅05, Fig. 3D), and provided independent prognostic value for further patient stratification (*P* = 0⋅002). Together, tumour EcoScore and mutational burden co-defined an aggressive subgroup of HGSOC with a high ecological imbalance and low mutational burden (low mutational burden, high EcoScore group vs. others: *P* = 2⋅5 × 10^−6^, HR = 2⋅25 [1⋅59–3⋅18], Fig. 3E).

### 3.4 Cancer adaptive strategy differs under specific ecological context

**Fig. 4:**
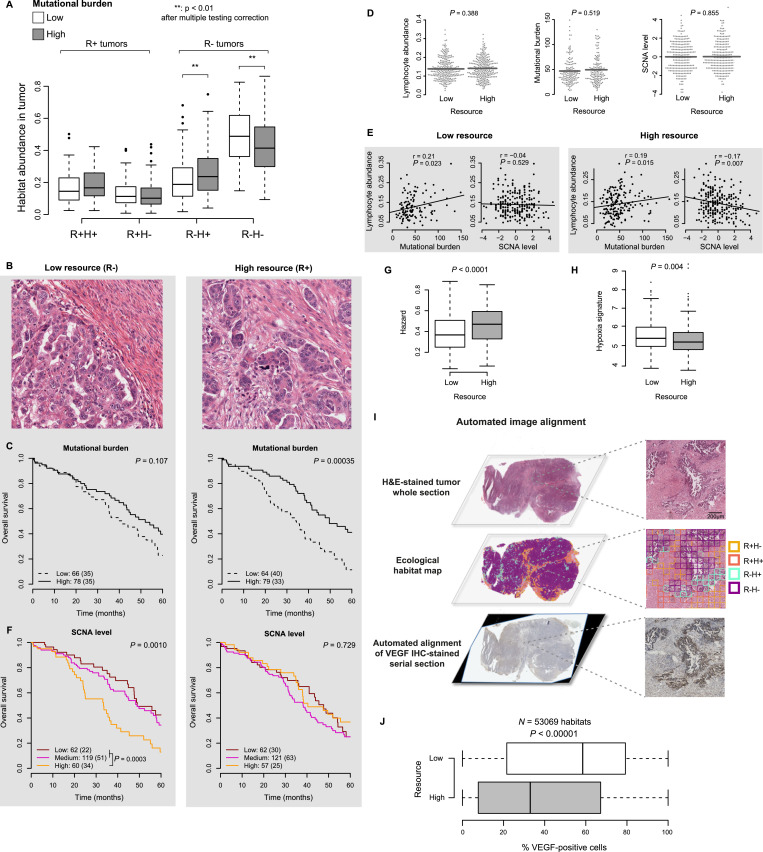
Different adaptive strategies in HGSOC based on ecological context. (**A**) Boxplots show the distribution of four ecological habitats in patients grouped by mutational burden (low: <45%); high: >45%). The R−H+/− habitats have a significant association with mutational burden as indicated by the two-sided Wilcox test p values. (**B**) Example H&E-stained tumour areas from a sample termed low (left) and high (right) resource. (**C**) Kaplan-Meier curves reveal mutational burden to be prognostic only in high resource tumours. Numbers outside parentheses indicate group size and numbers inside parentheses indicate deaths. (**D**) Swarm plots show no significant difference in lymphocyte abundance (left), mutational burden (middle) and SCNA level (right) between low and high resource tumours. (**E**) Scatter plots show mutational burden and lymphocytic abundance are positively correlated in both low and high resource tumours, but SCNA level correlates negatively with lymphocytic abundance only in high resource tumours. (**F**) SCNA level is prognostic only in low resource tumours. Numbers outside parentheses indicate group size and numbers inside parentheses indicate deaths. (**G**) Box plots show a greater abundance of high hazard habitats in tumours with high resource availability. (**H**) Box plots show a significantly higher mean expression of *CA9* and *VEGFA*, taken as surrogates for hypoxia, in low resource tumours. High and low resource is defined by the median value of high resource habitat abundance in this cohort. (**I**) A H&E-stained tumour region is shown (top row) along with corresponding ecological habitat classification (middle row) and VEGF protein expression (bottom row) obtained from automated image alignment of the VEGF IHC-stained section with the H&E-stained section from the same tumour. The low resource habitats (purple and cyan) spatially correlate with VEGF expression. (**J**) Boxplots show percentage VEGF positivity in a total of 53,069 low and high resource habitats obtained from four tumours from the Lan et al. cohort where VEGF IHC-stained sections were available. VEGF expression is significantly higher low resource habitats compared with high resource ones.

Since changes in mutational burden and genomic instability may have direct consequence in cancer fitness [42], we investigated how mutational burden and SCNA changed under different habitats to determine their selective advantage under different ecological contexts. We observed significant associations between mutational burden and the abundance of R− (R − H+/−) but not the R+ (R + H+/−) habitats, suggesting that stromal resource plays a role in immune response to neoantigens (Fig. 4A). Unlike mutational burden, SCNA, NtAI, LST and HRD-LOH were not found to correlate with the abundance of any ecological habitat (Supplementary Fig. S8A-D). However, when patients were divided into low (R − H+/−) and high (R + H+/−) resource groups based on our spatial definition of resource (Fig. 4B), the association between mutational burden and survival was only observed in the high resource group (Fig. 4C), regardless of cut-offs used within a reasonable range (Materials and Methods). On the other hand, SCNA, while also not different between low and high resource tumours (Fig. 4D), was found to be prognostic only among low resource tumours, where it was associated with significantly worse overall survival (*P* = 0⋅001, Fig. 4F).

In comparison, tumour-level stromal abundance did not provide the same spatial context for these genomic hallmarks as our resource definition. The prognostic value of SCNA was greatly reduced in the low stromal cell abundance group, with only a borderline significant difference in survival between patients with high SCNA and those with medium or low SCNA (Fig. S9A). There was no difference in the prognostic value of mutational burden when patients were grouped based on stromal cell abundance of the entire tumour (Fig. S9B). The prognostic values of other genomic aberrations (NtAI, LST and HRD-LOH) were not found to differ according to resource (Supplementary Table S2).

We then searched for differences between low and high resource tumours that may explain why mutational burden is prognostic in high resource tumours and SCNA in low resource tumours. There was no significant difference in overall lymphocyte abundance or the abundance of individual lymphocyte cell types as enumerated by CIBERSORT [43] (Fig. S10), nor in mutational burden between these groups (all *P* > 0⋅05, Fig. 4D). Both groups demonstrated a weak correlation between mutational burden and lymphocyte abundance, supporting the notion of tumour recognition by immune cells in response to tumour antigen [14] (*P* < 0⋅05, Fig. 4E). In contrast, lymphocyte abundance did not correlate with SCNA in the low resource tumours (Fig. 4E). Although they had a negative association in the high resource tumours, SCNA was not clinically relevant (Fig. 4E and F). Therefore, the prognostic value of mutational burden and genomic instability was ecological context-dependent, and cannot be explained by examining the abundance of lymphocytes and stromal cells alone. The weakness of the correlations found between the mutational burden and lymphocytic abundance may, in part, be a consequence of a difference in the specific locations of the tumour used for sequencing and image analysis. In the presence of tumour heterogeneity, such differences in sampling location are a likely confounders and are a limitation of the experimental design.

We next examined hazard, indicating co-localization of lymphocytes and cancer cells, in the context of tumour resource. Immune hazard was significantly higher in high resource tumours (*P* < 0.0001, Fig. 4G), suggesting that lymphocytes, while not differing in abundance between high and low resource tumours, exhibited greater co-localization with cancer cells in high resource tumours than low, thus presenting a more effective immune response following neoantigen recognition in these tumours. On the other hand, a hypoxia gene expression signature that included *VEGFA* and *CA9* [44] was significantly higher in low resource tumours (Fig. 4H). To validate this finding, automated alignment of VEGF IHC-stained tumour sections images to corresponding H&E images was performed to obtain the fraction of VEGF-positive cells in a total of 53,069 high- and low-resource habitats across validation samples (Supplementary Methods, Fig. 4I, Table S3). Low resource habitats had a higher fraction of VEGF-positive cells compared to high resource habitats (*P* < 0⋅0001, Fig. 4J).

## 4 Discussion

The evolutionary dynamics of neoplastic cells are shaped by the microenvironment that defines the selective pressures to which they are subjected [15,45]. In this study, we performed an integrative analysis of cancer evolutionary strategies and the microenvironmental context in which these strategies excel. Mutations in pathways such as DNA damage repair and DNA replication may initially increase the overall fitness of cancer, but a high mutational burden has been linked with the formation of neoantigens attracting immune predation and better patient survival rates [41,46]. In high-grade serous ovarian cancer which has the highest disease mortality among gynaecologic cancers, we evaluated how these key strategies of cancer perform under different microenvironmental conditions. By combining spatial and contextual histologic image analysis of the tumour microenvironment with genomics, we aimed to provide a powerful microenvironmental niche model to understand how the microenvironmental spatial complexity influences cancer adaptive strategies and progression.

The most significant finding from our study was that spatial architecture of tumour stroma could be a key determinant in successful adaptation of cancer to overcome microenvironmental constraints including immune surveillance and hypoxia. Spatial variation in resource distribution matters [47,48]. Patchy resources create multiple habitats, which may select for different clones and may respond differently to therapies. Here tumour resource was defined spatially, with high resource referring to tumours with a large number of endothelial cells and fibroblasts well distributed into close contact with cancer, potentially fuelling cancer growth and evolution. Whereas, low resource defines a spatial condition corresponding to absence or patchy distribution of stromal cells. Our data revealed that low resource tumours with high arm/chromosomal somatic copy number alterations (arm/chrom SCNA) are highly aggressive, despite SCNAs having no prognostic value in unselected ovarian cancer. In addition, the significantly higher hypoxia signature in low resource tumours compared to high resource tumours indicates that low resource tumours with high arm/chrom SCNA may be driven by rapid selection for alterations in cancer drivers. Genetic diversity of cancer cells as a result of aneuploidy and genomic instability provides large phenotypic variation and fuels evolution [49,50]. Under stress conditions, this has been shown to fuel rapid adaptive evolution, leading to selection [51]. Our data further indicated that, although SCNA appeared to be independent of immune abundance in low resource tumours, a lower level of cancer-immune co-localization may add to the ecological conditions favouring high SCNA. These results have significant implications for an important clinical consideration for evaluating the efficacy of emerging molecular therapies targeting detrimental aneuploidy, such as Hsp90 inhibitors [52], which is selecting patients most likely to respond to such therapies. We speculate that patients with low resource tumours, defined based on our spatial and contextual analysis of fibroblast and endothelial cell distribution, will be the most likely to respond.

Under the high resource condition, we discovered that low tumour mutational burden, but not high SCNA, confers advantages for cancer cells. Low mutational burden was strongly associated with decreasing lymphocytic infiltration estimated from histology. A high mutational burden can generate novel epitopes and gene products that can be recognized as foreign by immune cells [53], thus stimulating an anti-tumour immune response. While this is in line with our observation in the high resource tumours, in low resource tumours high mutational burden was no longer associated with prognosis. Given the consistent association between lymphocytic infiltration and mutational burden in both low and high resource tumours, we hypothesize that immune tracking of cancer is more effective in a high resource environment. In support of this, we found a significantly higher level of co-localization between lymphocytes and cancer cells, measured by our spatial definition of immune hazard, in high resource tumours. A speculation is that alterations to collagen or extracellular matrix create physical barriers in low resource tumours, manifest as patchy stroma, limiting effective immune surveillance by preventing T-cell trafficking into tumour nests [54,55].

The dependence of the prognostic value of mutational burden on the ecological context of stromal resource in our data is relevant given the consorted efforts underway to identify factors such as mutational/neoantigen burden for predicting immunotherapy response [56,57]. Our data also predict that this ecological context for resource may be useful, in combination with estimates of tumour mutational burden, for determining which patients are most likely to respond to developing immunotherapies to target neoantigens. We speculate that patients with high resource tumours will be best suited to personalized therapies such as neoantigen-specific T-cells or vaccines developed using neoantigen prediction following whole-exome sequencing of tumour samples [58]. Furthermore, understanding the mechanism by which stromal components affect anti-tumour immune responses may provide an avenue for further, novel therapeutic intervention. Therefore, the main novelty and differentiator from previous studies is that our ecological habitat classification reaches beyond a prognostic indicator: it represents biologically relevant cancer-microenvironment interplay that, together with cancer genetics, could unveil novel microenvironmental determinants of cancer evolution. Taken together, these data highlighted that both the evolutionary dynamics of the neoplastic cells themselves (cancer cell intrinsic factors), and the microenvironment that defines the ecology of those cells (cancer cell extrinsic factors), are important considerations in predicting the future behaviour and clinical response of a tumour [14].

The EcoScore was developed based on this foundation. A high EcoScore indicates favourable microenvironments for neoplastic cells with the ecological balance shifted towards resource-rich environments. A high EcoScore may be indicative of extensive stromal penetration of cancer cells. Our results showed that emphasis should be placed on local/short-range rather than global/long-range effects of resources and hazards on cancer cells for studying tumour ecology. The local EcoScore was associated with poor five-year overall survival in two independent cohorts of HGSOC patients with advanced disease, independent of clinical risk factors including residual disease after surgery, disease stage, age at diagnosis, response to treatment and *BRCA1/2* mutation, as well as lymphocyte and stromal cell abundance measures [24]. By combining EcoScore with mutational burden, we identified an aggressive subtype that may be the ideal patient subset for new therapeutics and highlighting opportunities for better classifications by combining cancer genetics and tumour ecology. In addition, we were able to identify a subgroup of high-risk patients despite optimal debulking surgery recognized as arguably the most important prognostic factor in ovarian cancer [59]. There is an urgent need to identify such patients [8], because although patients with optimal debulking are expected to have a better prognosis than patients with suboptimal debulking in general, a proportion will face more rapid disease progression, yet there is no available biomarker for their identification.

In summary, our study demonstrated that the ecological balance defined in the vicinity of cancer cells provides critical context for cancer cells to assume different evolutionary strategies, which motivated our development of a new prognostic marker independent of known clinical risk factors for HGSOC. The interaction between cancer cells and their surrounding healthy tissue is vital for tumour growth and evolution, yet they are often studied in isolation. Our study, although limited by access to large amount of validation tumour samples and being correlative in nature, provided new evidence supporting that the clinical consequences of cancer hallmarks, mutagenesis and aneuploidy, differ under specific tissue ecological contexts. Under high resource condition, selection may be driven by point mutations, whereas in low resource tumours, selection is fuelled by aneuploidy. Other limitations of our study include, firstly, that we used non-synonymous mutation count as an indicator of tumour mutational burden. This was due to non-availability of data on synonymous variants, although these are less likely to have an ecological impact since they do not lead to neoantigens and thus immunogenicity in the tumour. Second, our image analysis pipeline detects three cell types only, and cells such as macrophages and endothelial cells are not explicitly identified. With the rapid advances now occurring in computer vision, we expect this limitation will be adequately addressed in the near future. In addition, our findings could be consolidated by staining for identifying pro- and anti-tumour immune cells for further classification of immune hazards. Future work to understand the biological basis of fundamentally different tumour ecological contexts and how they alter the evolutionary trajectory of cancer through distinct mechanisms could pave the way for novel ecological therapies.

## Author contributions

S.N. contributed to study design, data acquisition following image processing of tumour histology, bioinformatics and statistical analyses, data interpretation and writing the manuscript; N.T. contributed to data acquisition by performing image registration and IHC analysis, statistical analysis, and writing the manuscript; A.H. contributed to data acquisition by performing tumour histology image analysis and validation; S.B. contributed to data interpretation and writing the manuscript; C.C.M. contributed to data interpretation and writing the manuscript; A.S. supervised the study; Y.Y. supervised the study and contributed to study design, data interpretation and writing the manuscript. All authors approved the final draft.

## Data availability

H&E-stained images from Lan et al., 2015 are deposited in the European Genome-phenome Archive under accession code EGAD00010000881. All clinical data required for reproducing the results in this study are included in the Supplementary Sweave file.

## Code availability

Available as a Supplementary Sweave file upon manuscript acceptance.

## CRediT authorship contribution statement

**Sidra Nawaz:** Conceptualization, Data curation, Formal analysis, Methodology, Visualization, Writing - original draft. **Nicholas A. Trahearn:** Formal analysis, Software, Validation, Visualization, Writing - original draft, Writing - review & editing. **Andreas Heindl:** Formal analysis, Software. **Susana Banerjee:** Investigation, Writing - original draft. **Carlo C. Maley:** Investigation, Writing - original draft. **Andrea Sottoriva:** Supervision. **Yinyin Yuan:** Conceptualization, Investigation, Methodology, Writing - original draft, Writing - review & editing.

## Declaration of Competing Interest

The funders had no role in the design of the study; the collection, analysis, or interpretation of the data; the writing of the manuscript; or the decision to submit the manuscript for publication. Y.Y. has received speakers bureau honoraria from Roche and is a consultant for Merck and Co Inc.

## Appendix Supplementary materials

Image, application 1
