# Supplementary material for: Analysis of tumour ecological balance reveals resource-dependent adaptive strategies of ovarian cancer
Source: eBioMedicine. 2019 Oct 21;48:224–35. doi: 10.1016/j.ebiom.2019.10.001 (PMC6838425; doi:10.1016/j.ebiom.2019.10.001)
Supplement: Supplementary file 1 [file mmc1.pdf]

Supplementary Methods

M1. Lymphocyte and stromal cell hotspot analysis

Our hotspot analysis pipeline was previously published in (60). The cell classification and location data was used as input for Getis-Ord hotspot analysis to enable the automated detection of statistically significant spatial clusters. Hotspot mapping is carried out on spatial data to identify locations where a variable of interest is found to be clustered. This means that, in relation to the entire area of study, the frequency or magnitude of this variable at these locations is greater than expected and, importantly, that the difference between the actual and expected value (determined from the area mean) is statistically significant. Tumor section images contain areas devoid of tissue, which were excluded from our analysis using a binary tissue mask constructed by CRImage (61) that classifies each pixel as tissue or non-tissue. Pixels corresponding to each region in the tumor were then summed up to determine the extent of tissue cover. Regions with less than 50% tissue cover were excluded from hotspot analysis. From the remaining regions, we also excluded those that contained no cancer cells, since our primary interest was to study tumor regions in close proximity to cancer.

To perform hotspot analysis on the tumor section images, a grid of square size s = 20 pixels/100μm and neighborhood size NR of 4 was used, as in (60), as input data is required in the form of spatial points with associated values and neighbors.

The z score for every region i is computed as,

$$z_i = \frac{\sum_{j=1}^{n} w_{i,j} c_j - \bar{c} \sum_{j=1}^{n} w_{i,j}}{SU},$$

where S and U are two normalizing factors, given by:

$$S = \sqrt{\frac{\sum_{j=1}^{n} c_j^2}{n} - (\bar{c})^2},$$

$$U = \sqrt{\frac{\left[n \sum_{j=1}^{n} w_{i,j}^2 - \left(\sum_{j=1}^{n} w_{i,j}\right)^2\right]}{n-1}},$$

where n is the total number of grid regions (excluding those with <50% tissue cover or no cancer cells); cj is the lymphocyte or stromal cell count for region j; $\overline{c}$ is the mean value of c for all regions in the image, and $w_{i,j}$ is element (i,j) of the matrix of weights, w, which indicates the influence of two regions on each other and is used to check whether or not the two regions are neighbors:

$$w_{i,j} = \begin{cases} 1 \; if \; j \; is \; a \; neighbour \; of \; i, \\ 0 \; if \; j \; is \; not \; a \; neighbour \; of \; i. \end{cases}$$

As in (60), a *z* score of 3·886 or above was taken to be a significant result, indicating statistically significant spatial clustering or hotspot formation.

M2. Immunohistochemistry staining

M2.1. CK7, CD8, CD3, CD20 and SMA immunohistochemistry staining

For a separate study(62), we used an automated pipeline to spatially align features obtained from image analysis of H&E sections with multiple serial immunohistochemistry (IHC) sections, using image registration and IHC image and spatial analysis. In brief, multiple sections were cut and placed in the same orientation on the slides, with the H&E sections midway through each series. The remaining sections were stained with CK7, CD3, CD8, CD20 and SMA, respectively. These were digitalized and spatially aligned to the H&E sections (detailed below). The accuracy of image registration was evaluated (average DICE coefficient = 0·91), and results were reported as a spatial correlation between IHC positivity and H&E image analysis results (average correlation CK7-cancer: 0·74, SMA-stromal: 0·70, CD3-lymphocyte: 0·67).

All staining was performed on the Leica Bond III platform using Leica Bond Polymer Refine Detection (Leica, DS9800). Blocking of endogenous peroxidase and non-specific staining was performed as per kit instructions. Following on-board dewax, and epitope retrieval (HIER) where necessary, primary antibody was applied for 15 minutes, followed by rabbit anti-mouse post-primary and anti-rabbit polymer for 8 minutes each, all at ambient temperature. Epitope retrieval was performed on-board at 99C using either Leica Epitope Retrieval Solution 1 or 2 (Leica, AR9961, AR9640; low and high pH solutions respectively): **CK7** (Leica, mouse clone RN7, cat. PA0138) used as supplied, HIER with ER2 for 20 minutes; **CD3** (Leica, mouse clone LN10, cat. NCL-L-CD3-565), used at a dilution of 1/100, HIER with ER2 for 20 minutes; **CD8** (Leica, mouse 4B11, cat.

PA0183) used as supplied, HIER with ER2 for 20 minutes; **CD20** (Agilent Technologies, mouse L26, cat. M075501-2) used at 1/100, HIER with ER1 for 20 minutes; **SMA** (Leica, mouse sm-1, cat. PA0943) used as supplied, no epitope retrieval required. Finally, the sections were counterstained with Mayer's hematoxylin, dehydrated, and mounted.

M2.2. VEGF immunohistochemistry staining

Four representative FFPE sections (Table S3) were randomly selected from the validation cohort by Lan *et al.*, 2015. 4μm thick tissue sections were dewaxed in xylene, and rehydrated through graded alcohol. After inhibition of the endogenous peroxidase and an antigen retrieval process, the sections were incubated with rabbit anti-human VEGF polyclonal antibody (ZSGB-BIO, Beijing, China, 1:50 dilution) for 50 minutes at 37°C. Primary antibodies were detected by EnVision kit (K5007, DAKO). The reaction was visualized using 3,3-diaminobenzidine (DAB). Finally, the sections were counterstained with Mayer's hematoxylin, dehydrated, and mounted.

M3. Image registration and scoring

IHC image registration was performed with a two-stage approach: an initial rigid alignment followed by a non-linear refinement. The first stage of the registration was performed by aligning the external boundaries of the tissue sections(63). The initial rigid registration was likely to be slightly inaccurate at high resolution, due to non-linear physical distortions that occur during sectioning. The registration corrected for this by performing a refinement procedure at high resolution to generate a non-linear registration transformation, based upon the initial rigid registration. The refinement was a local rigid alignment of salient tissue structures, such as nuclei clusters(64), and was performed on 500 x 500 pixel regions, sampled at a resolution of 0·46 μm/pixel. Coordinates of the corners of each region of interest were used as reference points to find the best-fit non-linear transformation. In this work a 4th degree polynomial transformation was used.

Automated scoring of the VEGF marker was performed on 100 μm x 100 μm regions of interest, sampled at a resolution of 0·46 μm/pixel. Regions were scored as the percentage of positive nuclei. Stain separation(65) was applied to the RGB image to extract the intensities of the hematoxylin and IHC stains. Manual thresholding was applied to the stain channels to identify regions of positivity. The proportion of positivity was estimated as the ratio

of the number of positive pixels in the IHC mask to those in the hematoxylin mask, with an upper cut-off of 100%. Other IHC markers were similarly scored as described in (64).

The regions selected for H&E classification were also used as the regions of interest for IHC scoring. These regions were mapped onto the IHC section using the transformation generated from the registration procedure outlined above.

References

[60] Nawaz S, Heindl A, Koelble K, Yuan Y. Beyond immune density: critical role of spatial heterogeneity in estrogen receptor-negative breast cancer. Modern Pathol 2015;28(6):766–77.

[61] Yuan Y, Failmezger H, Rueda OM, et al. Quantitative image analysis of cellular heterogeneity in breast tumours complements genomic profiling. SciTransl Med 2012;4(157) 157ra43.

[62] Heindl A, Khan AM, Nsudo Rodrigues D, et al. Microenvironmental niche divergence shapes BRCA1-dysregulated ovarian cancer morphological plasticity. Nat Commun 2018;9(1)) under review.

[63] Trahearn N, Epstein D, Snead D, Cree I, Rajpoot N. A fast method for approximate registration of whole-slide images of serial sections using local curvature. SPIE Med Imag Digit Pathol 2014;9041:90410E.

[64] Trahearn N, Epstein D, Cree I, Snead D, Rajpoot N. Hyper-Stain inspector: a framework for robust registration and localised co-expression analysis of multiple whole-slide images of serial histology sections. Sci Rep 2017;7(1):5641.

[65] Trahearn N, Snead D, Cree I, Rajpoot N. Multi-class stain separation using independent component analysis. SPIE Med Imag Digit Pathol 2015;9420:94200J.

Supplementary Figures

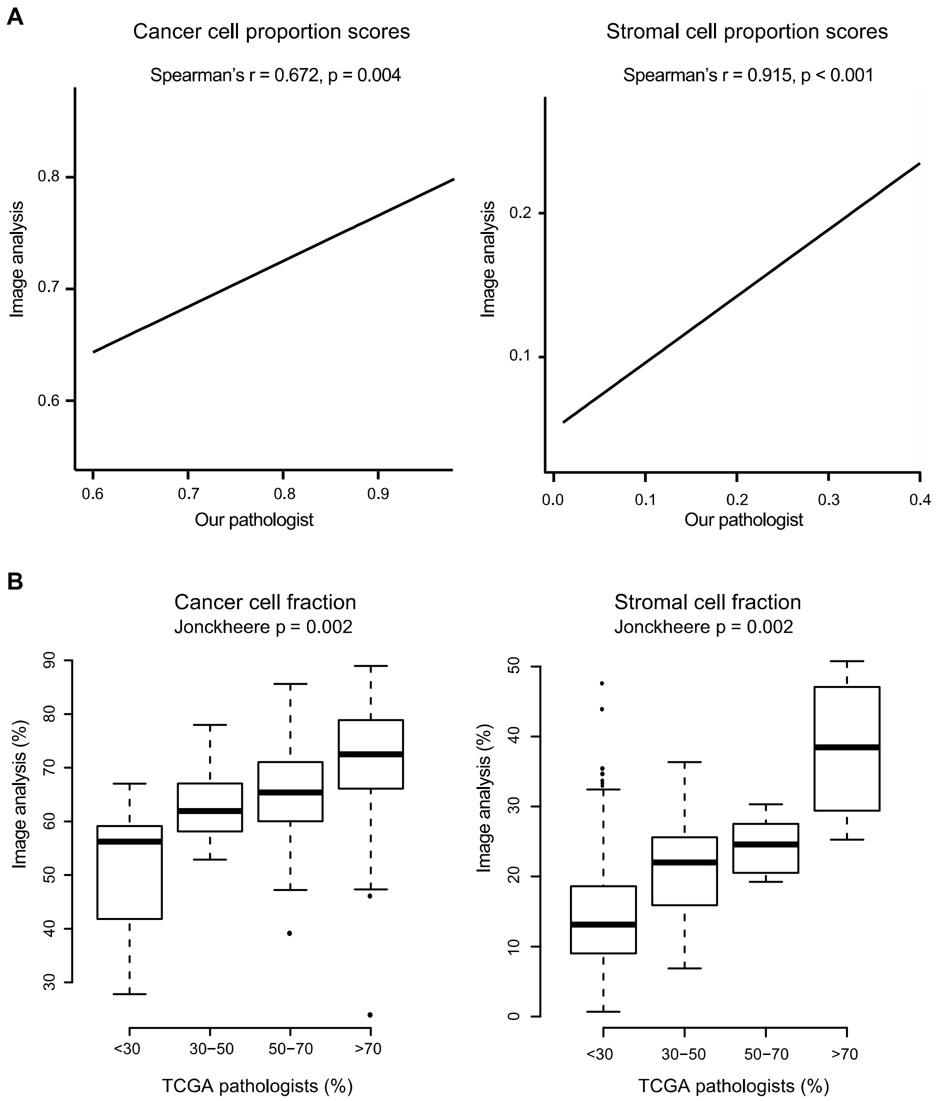

Fig. S1. A. Comparison between cancer (left) and stromal (right) cell proportions as visually assessed by our pathologist and automated quantification by our image analysis pipeline adapted for analysis of histology slides obtained from frozen HGSOC tumors. **B.** Comparison between cancer (left) and stromal (right) cell fractions as reported by pathologists from TCGA in four categories and automated quantification by our pipeline.

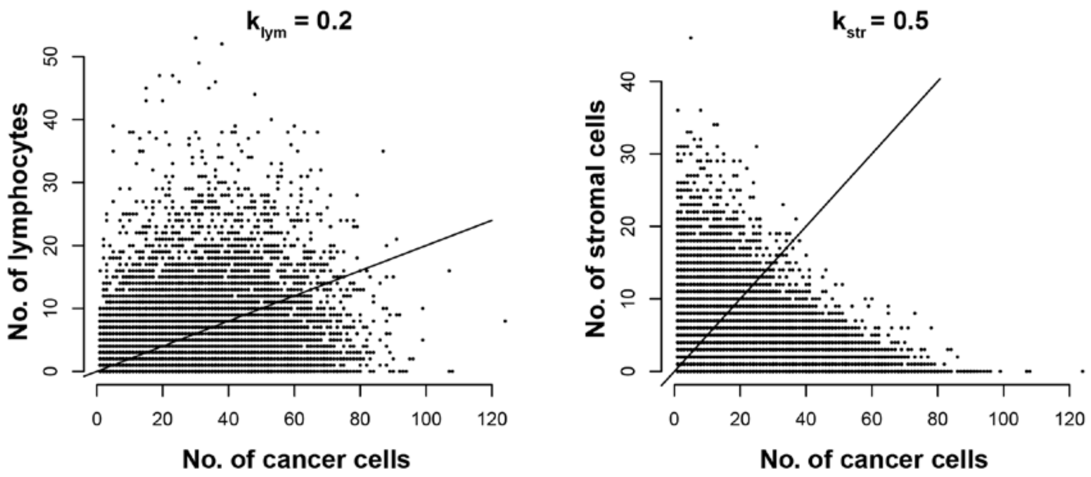

Fig. S2. Choosing a threshold to define high and low lymphocyte (left) and stromal cell (right) abundance in order to characterize a region into one of the four ecological niches. Each point indicates one of a total of 100,000 100μm square regions randomly sampled from 100 randomly selected tumors in the discovery cohort. For lymphocytes, the gradient of the diagonal line was chosen in order to split the number of regions such that half are classified as having high numbers of lymphocytes and half low. For stromal cells, the gradient was chosen such that only 25% of the regions were classified as having a high stromal abundance relative to cancer cells. The line thus defines the minimum relative abundance of lymphocytes (20%) and stromal cells (50%) to cancer cells that a region must have in order to be classified, respectively, as high lymphocyte (hazard) and high stromal (resource).

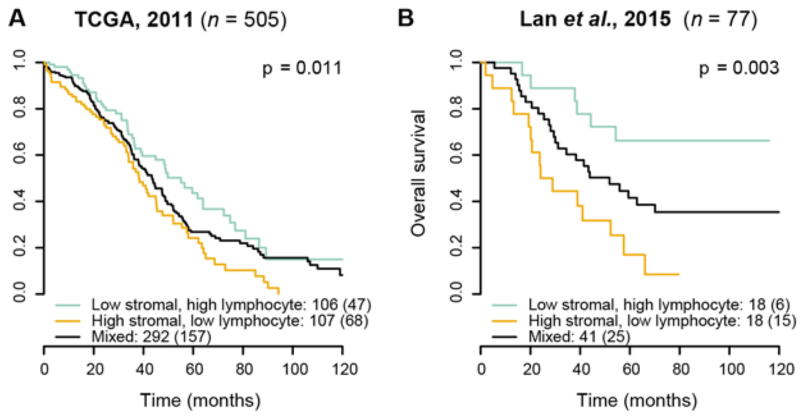

Fig. S3. Kaplan-Meier curves illustrating 10-year overall survival of patients from two independent HGSOC cohorts, TCGA, 2011 (**A**) and Lan *et al.*, 2015 (**B**), stratified by lymphocytic and stromal cell abundance as enumerated from histology image analysis. A high lymphocytic abundance and a low stromal cell abundance correlates with good prognosis. Numbers outside parentheses indicate group size and numbers inside parentheses indicate deaths.

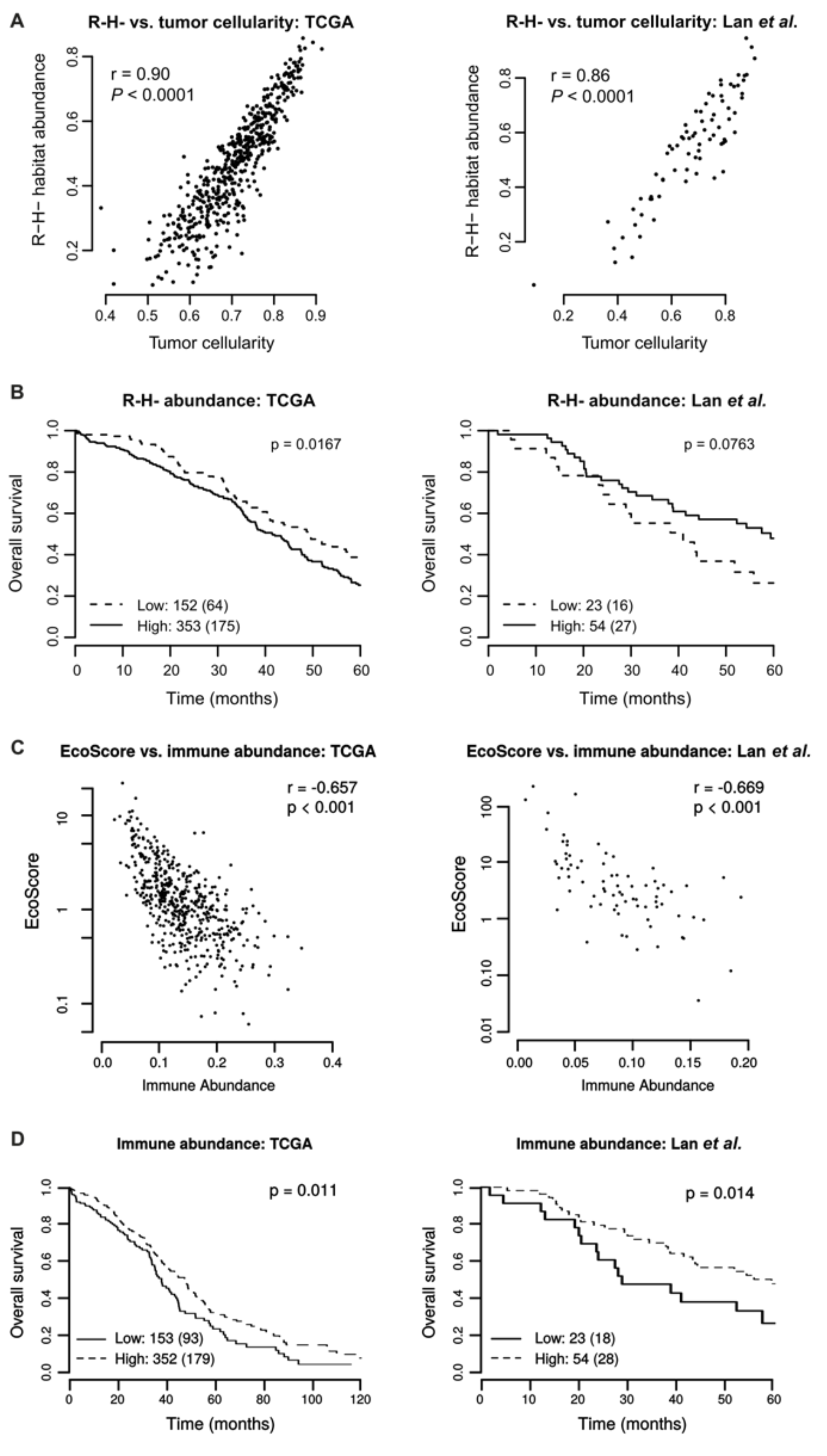

Fig. S4. A. Scatter plots display the strong positive association between R-H- abundance and tumor cellularity in the two cohorts. **B.** Kaplan-Meier curves display the overall survival of patients stratified using an optimum threshold for R-H- abundance in the TCGA and Lan *et al.* cohorts. The association changes direction in the Lan *et al.* cohort, making the prognostic value of R-H- abundance unclear. **C.** Scatter plots display the negative association between EcoScore and lymphocytic abundance in the two cohorts. **D.** Kaplan-Meier curves display the overall survival of patients stratified using an optimum threshold for immune abundance in the two cohorts.

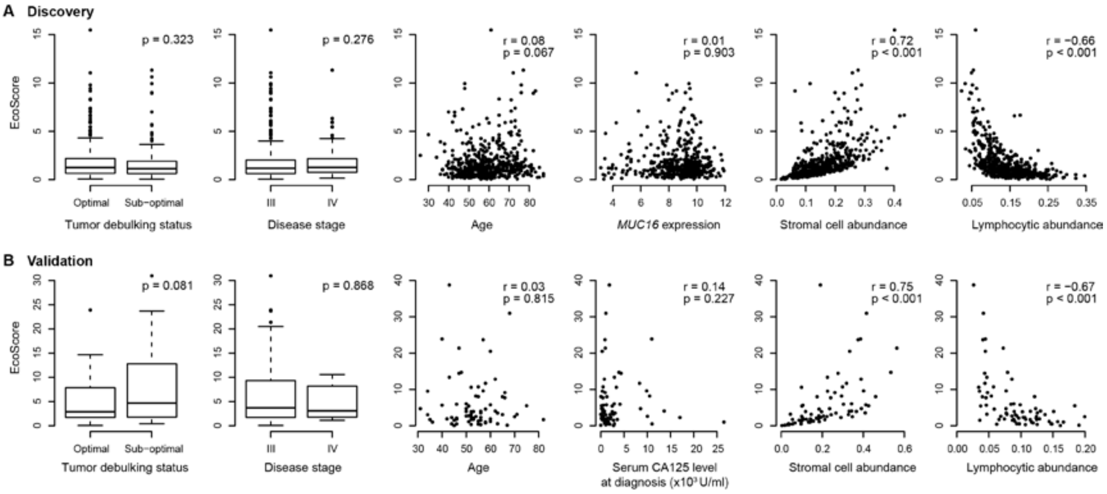

Fig. S5. The relationships between EcoScore and known clinical risk factors for ovarian cancer, as well as cell abundance measures, in the discovery cohort from TCGA, 2011 (**A**) and validation cohort from Lan *et al.*, 2015 (**B**) are shown. Lymphocytic and stromal cell abundance are significantly correlated with EcoScore.

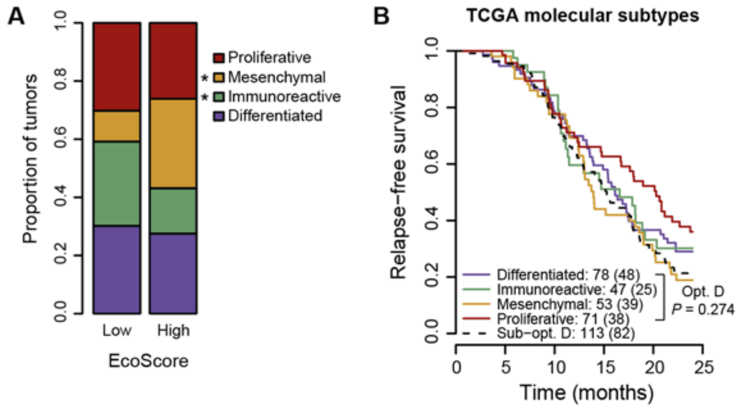

Fig. S6. A. Barplots show the differences in four HGSOC molecular subtypes between low and high EcoScore tumors. *: two-sided Wilcoxon test p value < 0·0001. **B.** Stratification using molecular subtypes of patients in the discovery cohort who had optimal debulking surgery (colored curves). The stratification was not prognostic. Numbers outside parentheses indicate group size and numbers inside parentheses indicate deaths.

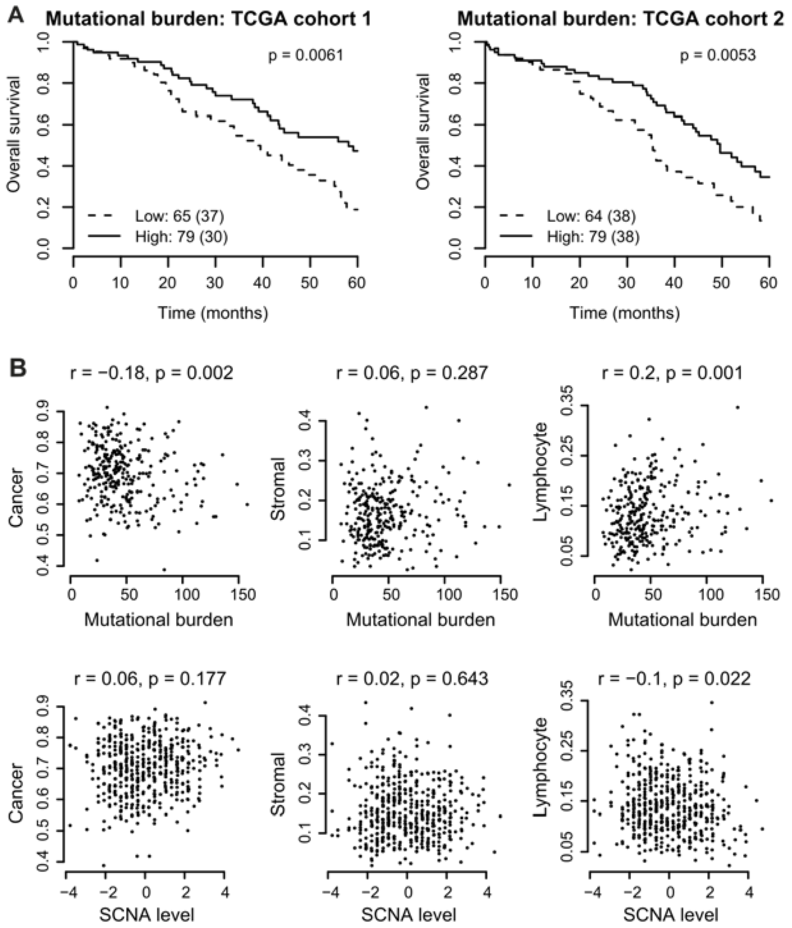

Fig. S7. A. Kaplan-Meier curves display the overall survival of patients stratified by mutational burden in the TCGA cohort. The cohort was randomly divided into two groups and an optimum threshold in mutational burden was found in cohort 1 and validated in cohort 2 over 10 repeats. **B.** Scatter plots display the association of SCNA level and mutational burden with cancer cell, stromal cell and lymphocytic abundance.

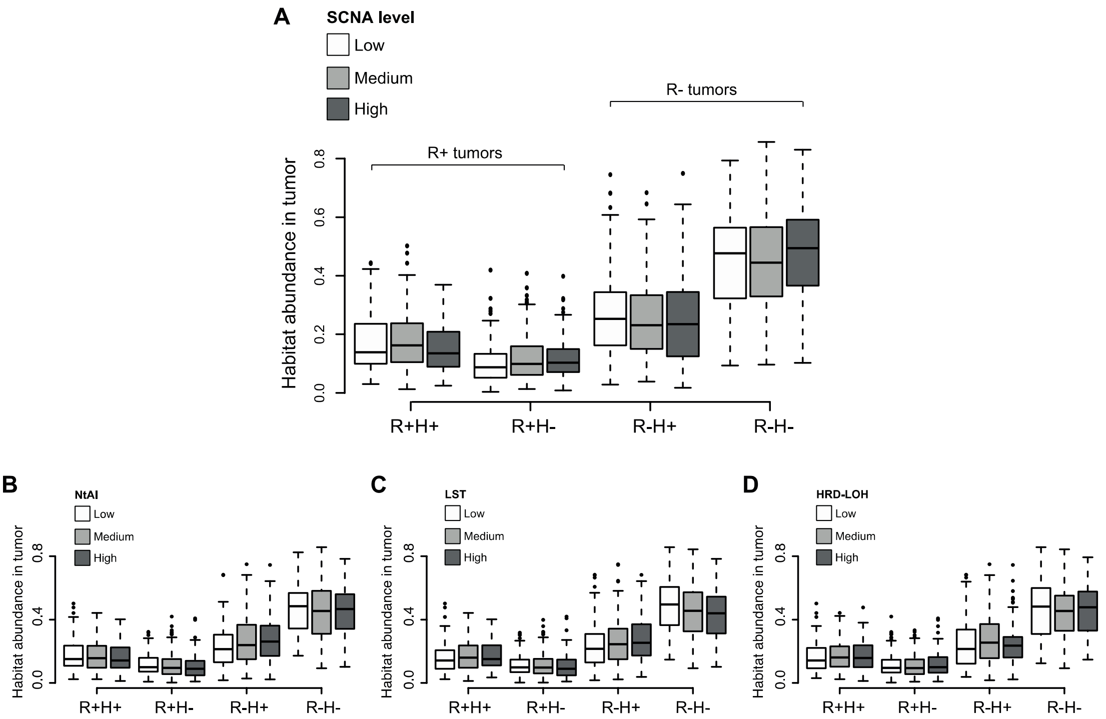

Fig. S8. Comparison of ecological habitats to genomic measures. Boxplots show the distribution of four ecological habitats in patients grouped by SCNA level (**A**), number of telomeric allelic imbalances (NtAI, **B**), large-scale transitions (LST, **C**) and loss of heterozygosity (HRD-LOH, **D**). None of the habitats associate significantly with any of these genomic measurements (all $P > 0{\cdot}5$). Low: <25%; medium: 25–75%; high: >75%.

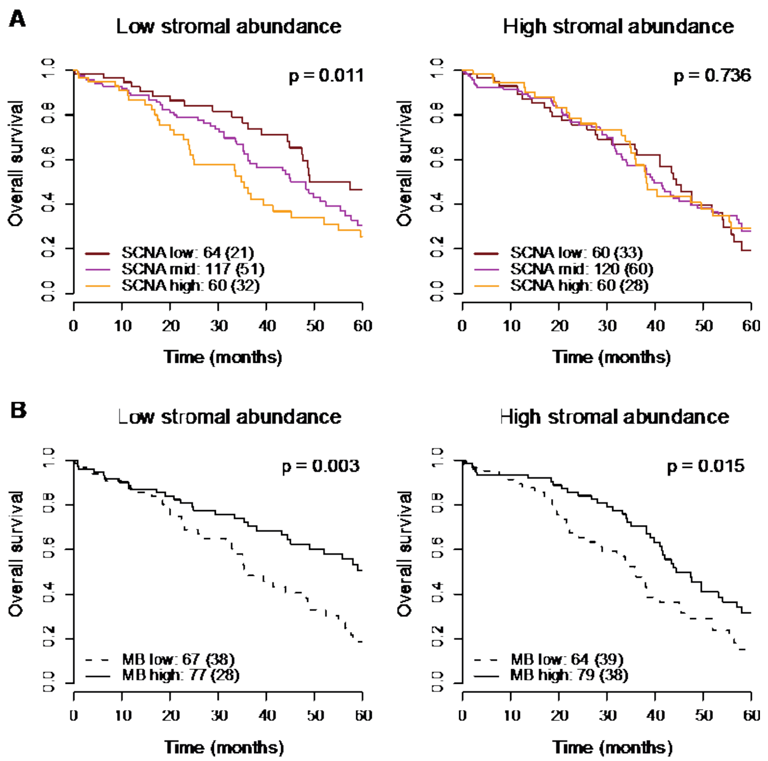

Fig. S9. A. Kaplan-Meier curves show the prognostic value of SCNA level in tumors with low (left) and high (right) stromal abundance. SCNA level is prognostic if tumors have a low stromal abundance, but its prognostic power is much lower compared with when the spatial context of stromal cell distribution is taken into account (Table S2). **B.** Kaplan-Meier curves show the prognostic power of mutational burden (MB) in tumors with low (left) and high (right) stromal cell abundance. The prognostic value of MB is statistically significant in both groups. Numbers outside parentheses indicate group size and numbers inside parentheses indicate deaths.

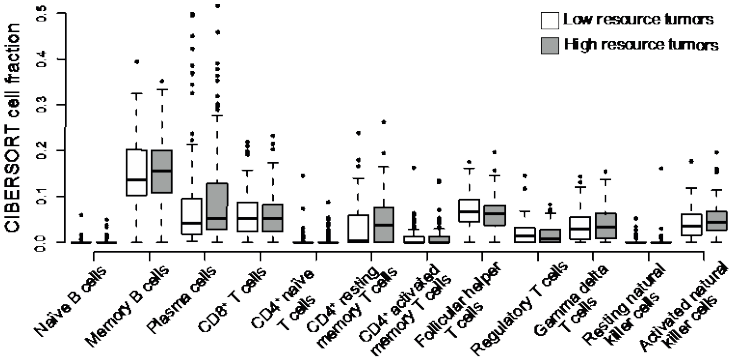

Fig. S10. CIBERSORT analysis estimating the fractions of 12 subtypes of lymphocytes based on mRNA expression show no significant differences in any of these subtypes between low and high resource tumors.

Supplementary Tables

	Discovery (*n* = 505)		Validation (*n* = 77)	
	Low EcoScore	High EcoScore	Low EcoScore	High EcoScore
**Number of patients**	202	303	31	46
**Median 5-year overall survival (months)**	52·0	38·1	N/A	38·3
**% Received platinum compound**	100		100	
**% Received a taxane**	94		88	
**% Response to primary treatment**				
**Complete response**				
**Partial response**	74·8	66·8	N/A	
**Stable disease**	10·5	17·1		
**Progressive disease**	7·7	6·5		
	7·0	9·7		
**% Platinum sensitivity**				
**Sensitive**	70·9	67·1	N/A	
**Resistant**	29·1	32·9		
**% *BRCA1/2* mutation**				
**Wildtype**	77·0	77·4	N/A	
**Mutant**	23·0	22·6		
**Mean CA125**				
**Serum level at diagnosis (U/ml)**				
**Expression (*MUC16*, AU)**	N/A	N/A	3290	2770
	8·56	8·69	N/A	N/A
**% Debulking status**				
**Optimal**	68·9	71·0	64·5	52·2
**Sub-optimal**	31·1	29·0	35·5	47·8
**Mean age at diagnosis (years)**	58·9	60·1	53·1	53·8
**% Disease stage (FIGO)**				
**III**	87·1	81·8	87·1	89·1
**IV**	12·9	18·2	12·9	10·9
**Mean stromal cell abundance (%)**	10·3	19·1	12·1	30·6
**Mean lymphocytic abundance (%)**	17·5	11·6	11·5	7·2

Table S1. Patient summary statistics for the two HGSOC cohorts: discovery from TCGA, 2011, and validation from Lan *et al.*, 2015, split using our strongest prognostic marker, the EcoScore. Listed in the table are survival rates for all groups as well as data for known risk factors for HGSOC that are available for the cohorts. Median survival is taken as the time until 50% of the sample has undergone the event. In the validation cohort, less than half of patients with a low EcoScore died within five years of diagnosis, hence an N/A is given. Data for serum CA125 level at diagnosis and type of chemotherapy each patient received are not available for the validation cohort from TCGA. Data on response to primary treatment, platinum sensitivity, *BRCA1/2* mutation and *MUC16* expression were not available for the discovery cohort. AU: arbitrary units.

	**All tumors**	**R+**	**R-**
**SCNA level**	*P* = 0·056, poor prognosis	*P* = 0·729	*P* = 0·001, poor prognosis
**Number of telomeric allelic imbalances**	*P* = 0·401	*P* = 0·606	*P* = 0·474
**Large-scale transitions**	*P* = 0·009, good prognosis	*P* = 0·008, good prognosis	*P* = 0·049, good prognosis
**Loss of heterozygosity**	*P* = 0·558	*P* = 0·331	*P* = 0·025, good prognosis

Table S2. The prognostic value of four genomic measures [1, 2] for five-year overall survival of patients in the TCGA cohort. The overall prognostic value as well as separately for high (R+) and low (R-) resource tumors is given. Where differences in patient survival are significantly different according to the scores, the direction of association is also given.

	Sample 1	Sample 2	Sample 3	Sample 4
**Debulking status**	Sub-optimal	Sub-optimal	Sub-optimal	Sub-optimal
**Disease stage**	III	III	III	III
**EcoScore**	High	High	High	High
**Lymphocytic abundance %**	7·2	10·2	7·8	8·4
**Stromal abundance %**	56·3	19·7	26·7	30·4
**Overall survival (months)**	25·0	34·0	16·0	59·4

Table S3. Clinical risk factors, cell abundance measures and the EcoScore are given for the three samples for which VEGF immunostaining and image alignment were performed to obtain VEGF expression scores per habitat.
